# Peridental Anesthesia

**Published:** 1912-12-15

**Authors:** Frank L. Platt

**Affiliations:** San Francisco, Cal.


### PERIDENTAL ANESTHESIA.

BY FRANK L. PLATT, D.D.S., SAN FRANCISCO, CAL.

The interior of the alveolar process between the outer and inner plates is composed of what is known as cancellous tissue, a spongy mass of bone containing numerous little spindle-shaped spaces called lacunae, branching out from which in various directions are minute channels called canaliculi, which anastomose with similar canals from other lacunae. The interstices of the cancellous tissue are filled with marrow, which in the maxillae is of the red variety.

The alveolar process is covered with periosteum the blood-vessels and nerves of which pass through the canaliculi and communicate with the marrow.

The roots of the teeth are surrounded with the peridental membrane, a connective tissue well supplied with nerves and blood-vessels and continuous with the periosteum covering the outer alveolar walls. It affords nourishment to the bone on one side and the cementum on the other, through its fibrous elements holds the teeth in their alveoli and through its abundance of nerves acts as the external organ of sensation of the teeth.

The nerve and blood supply of the peridental membrane is derived from the blood-vessels and nerves entering the apical portion of each alveolus where they divide, some entering the apical foramen to supply the dental pulp and others passing into the peridental membrane unite with the vessels of the periosteum through the alveolar process.

It will thus be readily seen that if the peridental membrane is rendered insensitive by the application of an anesthetic injected either directly into it or onto the cancellous portion of the alveolar process the entire tooth may be anesthetized owing to the close relationship of the nerves and blood-vessels supplying the pulp and peridental membrane.

The dangers attending the use of cocain, and the fact that it has no known antidote, have led numerous investigators to seek a substitute for it, and many have been placed before the profession, including tropococain, the eucains, and novocain, the latter now being generally used as the best and safest substitute.

The toxicity of novocain is from two to six times less than that of cocain; it causes no irritation when injected and will combine in any proportion with adrenalin the latter being used to localize the effect of the anesthetic.

In such a combination we have an ideal preparation for inducing peridental anesthesia.

The necessary equipment for this procedure consists of an all-metal hypodermic syringe; clean, sharp 28-gauge needles, the needles extending beyond the reinforcement from 3-16 to 1/4 of an inch, and for the intraosseous injection where the process is punctured by a drill or bur, a conical needle, which may be made by cutting the steel needle off at the point of reinforcement and grinding the latter down to a long, tapering point; a glass graduate for mixing the anesthetic; clean cotton rolls or napkins, and an atomizer for cleansing the gums and mucous membrane of the oral cavity.

With the atomizer filled with a solution of any good antiseptic mouth wash the gums are cleansed as well as possible, particular attention being paid to the region about the necks of the teeth under the free border of the gums, as it is here that sterilization is most essential and most difficult to accomplish. In patients under fourteen years of age the peridental membrane may be readily reached by inserting the needle into the interdental flap, on which pressure may be applied with the finger to render the first puncture of the needle painless, then with light pressure on the piston of the syringe to keep the anesthetic always ahead of the needle, force the needle into the tissue until the interdental septum of bone is reached; then deflect the needle at an angle of about fifteen degrees with the long axis of the tooth toward the tooth to be anesthetized and force the needle directly into the peridental membrane. At no times should heavy pressure be used as it may lead to leakage of the anesthetic, or rupture of tissues with subsequent peridental inflammation.

In older patients where the peridental membrane is thinner and the process harder the needle should be driven directly into the cancellous tissue of the interdental septum with light blows on the head of the piston of the syringe, stopping at frequent intervals to grip the syringe to determine if the needle has become clogged.

If this happens, remove the needle and with heavy blows on the piston remove the obstruction, reinsert the needle in the same puncture and proceed until the outer plate of bone is punctured and the cancellous tissue reached.

Here a direct venal injection is made and the anesthetic should be deposited in the tissue very slowly, from one to two minutes consumed to deposit twenty minims.

In cases where the process is too hard or dense to be penetrated by the needle, a point on the gum about half way between the apex ot the root and the gingival border is selected, either between the teeth, or in the case of a molar, between the roots of the tooth and one or two minims of the anesthetic is injected; then holding the lip or cheek well out of the way and in a fixed position so the tissue will not slip about over the alveolar process, with a number one-half round bur, sterilized and dipped in carbolated vaseline, drill through the outer plate of the process, first pressing the bur through the gum before starting the engine to avoid twisting the tissue on the bur.

Having replaced the hypodermic needle with the conical needle, insert the latter in the hole in the process and inject slowly as in cases where the needle is driven into the cancellous tissue. Particular care should be used to hold the gum firmly in position, for if it moves at all it is sometimes difficult to locate the point of the puncture with the conical needle and the tissues may be badly lacerated trying to do so.

After anesthesia has been affected, and the operation completed, the atomizer should again be used and the gum thoroughly massaged to aid in re-establishing the circulation.

In no case should the needle be inserted under the free margin of the gum, as it will almost certainly result in infection and it is well to explore this region carefully with a small blunt probe to determine the width of the free margin of the gum and the exact point of gingival attachment.

This is particularly true in pyorrheal conditions. Excepting in the case of children, where the peridental membrane is easily reached I have found the alveolar puncture method, that is where the process is penetrated with the bur, the safest and most satisfactory.

There is less danger of infection, particularly in cases of gingivitis or pyorrhea, and anesthesia is quickly and readily accomplished. Experience is the best guide for the selection of the method to be employed and one soon learns by the appearance of the gums, and the age and temperament of the patient which one to select.

Peridental anesthesia solves the problem of painless dentistry, for by its use any operation in dentistry may be performed painlessly; cavities may be prepared, pulps removed or teeth extracted without infliction, and should sensation return to the tissues before the operation is completed a second or third injection may be made.

These are seldom required however, as the initial anesthesia lasts twenty to forty minutes.

Peridental anesthesia is in my opinion one of the greatest achievements of dental science, as it renders possible the thorough performance of dental operations now frequently imperfectly executed owing to the limitations of human endurance on the part of both patient and operator, banishes fear, the prime factor leading to personal neglect of the teeth, and lessens in the most effective manner possible the nervous strain incident to the careful performance of dental operations.

With the adoption and development of this method of anesthetizing the tissues on which we operate, I believe, to quote from an editorial in the *Pacific Dental Gazette*, "The day is not far distant when the dentist who inflicts pain in the practice of his profession will be looked upon much as a surgeon would be today should he strap his patient to the operating table rather than resort to the use of a general anesthetic."

DISCUSSION.

Dr. R. C. Baker: I am glad the essayist has mentioned novocaine. As I understand it, it may be kept sterile by boiling, without losing its properties. He speaks of using this in all dental operations. In my work in pyorrhea, I object seriously to the injection of anything of this kind into the diseased tissues. They are at a low state of vitality swollen and very sore and it seems useless to inflict them further with any injection. After the operation for pyorrhea, the tissues have enough to do to recover themselves. Another objection in anesthetizing the peridental membrane that the operator, unless very careful indeed, would inflict a great deal of injury to this membrane and the tissues about the tooth for the reason that the patient is no help to him as to how deep he is going and he may injure unnecessarily the peridental membrane. A careful operator may work without inflicting more pain than when injecting the anesthetic. I have noticed an article by Dr. Wright of the London General Hospital. He speaks of novocaine and of injecting the drug into the spongy bone. He states the danger in using the new drug is only one-third as great as in using cocain. He says dependence upon local anesthetic is more common there than in the United States. In Norway the new anesthetic is so popular that the extraction of teeth with general anesthesia is now regarded as unnecessary.

Dr. C. E. Fox: I have been following this method for two or three years. I have had some great successes and some dismal failures. One thing I would like to bring out is with reference to being guided as to how far to put the bur in going through the process. One should be guided by the way the bit feels as it goes through. Some processes are very thick and some are very thin. It feels as when going in the pulp chamber, for instance. Be careful as to the line in which the bit is held so as to guide it through between the teeth instead of striking the teeth. Much damage may be done, as the doctor says, by striking the peridental membrane. I prefer this method over peridental injection, except in children, because of the resistance offered by the peridental membrane.

Dr. Flood: In discussing this question of surgery with a physician friend a short time since, he advised me that many surgeons today are performing even capital operations and where the sutures are inserted—the wound closed and rendered sterile—the last thing done is to wash the wound, sutures and all, with a strong solution of iodine. It seems to render it absolutely sterile, and it is then sealed absolutely with wax, etc., for seven days and usually there is no pus as heretofore. I have followed that practice in peridental anesthesia and after cleansing the gum previous to inserting the needle I wet the gum with iodine and I have had no pericementitis since using that method.

Dr. Platt: In clean and healthy mouths, I do not often use the tincture of iodine. In dirty mouths, I do use it. I think it is a safeguard which it will be well to employ. I would say the anesthetic can be injected absolutely without pain. Always keep the anesthetic ahead of the needle. I have seen men use the anesthetic where they put the needle from ½ to ¾ inch into the patient before injecting. Always cleanse the gums; use 28-gauge needle; have a fresh needle every time. The minute you press the bevel of the needle against the gum, begin to inject; keep the anesthetic ahead of the needle and little children will not shrink from the operation in any way. I do not tell my patients what I am going to do. If removing a pulp, do not mention that fact. A patient came to me with a lower molar in such a condition that I felt the pulp must be removed. I did not tell the patient what was going to happen. She has always been a little apprehensive. I went ahead, made my injection, opened up the tooth, removed the pulp, and put in a temporary filling. Then she asked, "Doctor, do you have to take the nerve out of the tooth?" I said, "No it has been out fifteen minutes already."—*Pacific Gazette*, Nov. 1912.

